# Analysis of Influencing Factors and Mechanism of Farmers’ Green Production Behaviors in China

**DOI:** 10.3390/ijerph20020961

**Published:** 2023-01-05

**Authors:** Zejun He, Yunfei Jia, Yifan Ji

**Affiliations:** 1 College of Economic and Management, Henan Agricultural University, Zhengzhou 450002, China; 2 Management School, Hainan University, Haikou 570228, China

**Keywords:** green production behaviors, influencing factors, action mechanism, small- and medium-sized pig farmers

## Abstract

The green production behavior of pig farmers is the basis for high-quality development in animal husbandry. In order to solve the problem of poor green production behaviors in small- and medium-sized pig farmers, it is necessary to analyze the influencing factors and how they interact with each other. The Rational Peasant Theory and Prospect Theory were used in this paper to analyze the occurrence motivation of the green production behaviors of small- and medium-sized pig farmers. The Logit model and the ISM analysis method were used to test the influencing factors and their mechanisms. This was conducted using data from a study of 747 small- and medium-sized pig farmers in Henan Province. The results show that the green production behaviors of small- and medium-sized pig farmers are motivated by internal expected return, affected by the monitoring pressure from external stakeholders and limited by their own resource capacity; the influencing factors of different green production behaviors are different, and there are more influencing factors of scientific disease control, standardized management and waste recycling than of rational feeding. The following shows how the influencing factors on pig farmers’ green production behavior interact with one another: level of education → external pressure, farming conditions and operating characteristics → cognition of return → green production behavior (i.e., cognition of return is the direct factor; external pressure, farming conditions and operating characteristics are indirect factors; and level of education is the underlying factor). Some measures should be implemented to promote green production behaviors, such as the continuation of the support for green production, the strengthening of supervision and publicity, the increasing of investment in technology and equipment, and the improving of the green production literacy of farmers. In conclusion, this paper deepens the understanding of the mechanism of green production behaviors of small- and medium-sized pig farmers, and provides the theoretical basis and concrete measures for the government and for pig farmers.

## 1. Introduction

Pig farming must follow the path of sustainable development. According to the Decision on Promoting the High-quality Development of Animal Husbandry of the General Office of the State Council (G.B.F. [2020] No. 31), it is required to foster a new pattern of high-quality development of animal husbandry featuring efficient output, product safety, resource conservation, environmental friendliness and effective regulation. It is also helpful to keep raising the bar of the industry’s quality, productivity and competitiveness. To grow animal husbandry in a high-quality manner, pig farming must first advance environmentally friendly development. This is due to the fact that pig farming is a key sector of China’s animal husbandry and that more than 60% of total meat consumption is from pork. On the other hand, pig farming is responsible for more than 40% of all pollution from livestock and poultry farms [1]. Concern is also growing over the safety of pork products [2].

The green development of pig farming cannot be advanced if small- and medium-sized farms do not adopt green production practices. It is farmers that actually put green production into practice. Their production behaviors have a direct impact on the quality of pork and whether pig farming pollutes the environment, which also decide whether or not the sector develops sustainably. More than 98% of all pig farmers are small- to medium-sized pig farms or individual pig farmers, with farming stock making up more than half of the total. The biggest challenges to green development are found on small and medium-sized farms and are due to insufficient epidemic prevention, challenging product quality monitoring and inappropriate manure treatment [3]. The application of pertinent government policies by small- and medium-sized farmers has not yielded satisfactory results, despite the fact that there have been numerous policies on the green growth of pig farming since the 18th National Congress of the CPC [4]. As China’s economy moves into the stage of high-quality development, it will be very important from a practical standpoint to investigate how to encourage small- and medium-sized farmers to adopt environmentally friendly production practices.

Numerous studies have been conducted on the factors that contribute to farmers’ adoption of environmentally friendly production practices, but not enough has been done to understand how small- and medium-sized farmers actually behave in this way. Farmers’ knowledge of green production, environmental risks, clean and safe production, green production policies, and economic and social benefits is a significant contributor to their green production behaviors [5,6,7,8]. Government rules, social standards and environmental laws can all motivate farmers to adopt green production behaviors [9,10]. Industrial cooperation organizations could hasten the adoption of green production behaviors by promoting technology, standardizing management and dispersing operational risks [11,12]. Many scholars have held the opinion that the operations and individual characteristics of farmers have a significant bearing on their approach to sustainable production [13,14]. Some scholars believe that the implementation of green production behaviors by farmers is the result of a combination of factors, such as usefulness perception, technical training, technical service organization and economic and technological subsidies [15,16,17]. The existing literature has empirically analyzed predominantly the influencing factors of green production behaviors, and the results serve as valuable source for this paper. However, there are also issues that need to be addressed. First, analysis of the mechanism of green production behaviors is insufficient. Farmers’ green production practices must meet a number of requirements, since they have a positive externality effect. But the existing studies hold that it is mainly the result of objective conditions, and the analysis of subjective anticipation factors is insufficient. Second, series of green production behaviors have not been thoroughly studied. A number of farmers’ green production practices have culminated in the development of green farming. The existing studies have examined the factors contributing to a particular green production behavior of farmers, but less attention has been paid to a series of green production behaviors. Third, the analysis of the interactions between the elements that influence green production behaviors is lacking, yet these interactions define how those behaviors are affected. The studies that are now available focus more on the evaluation of the impact of various factors and less on how those factors interact.

Based on the analysis above, this study examines the following issues regarding the green production practices of medium- and small-sized pig farmers: (1) What drives medium and small-sized pig farmers to adopt green production behaviors? (2) What elements sway pig farmers to adopt sustainable farming practices? Are there differences in the influencing factors of different green production behaviors? (3) How do the factors interact with each other? After defining the connotation and analyzing the occurrence motivation of green production behaviors, this paper explores the influencing factors of green production behaviors and the interaction mechanism between them, respectively, using the survey data of small- and medium-sized pig farmers in Henan Province with the Logit model and ISM (Interpretative Structure Model) analysis method, in order to put forward corresponding theoretical guidance and policy recommendations for the green development of pig farming and high quality development of animal husbandry in China.

## 2. Concept Definition, Theoretical Analysis and Hypotheses

### 2.1. Definition

Green production behavior is a production mode that can not only guarantee and boost agricultural productivity and profitability, but also improve resource utilization efficiency and reduce environmental pollution in rural areas [18]. Based on this concept, the connotation of green production behavior includes at least three dimensions: high efficiency of output, resource saving and environmental friendliness. If the social value of the behavior is considered, product safety is also one of the important dimensions. The four dimensions are consistent with the goals of high output efficiency, product safety, resource conservation and environment friendliness in the high-quality development of China’s livestock industry. The difference is that green development is defined by the operating entities at the micro level, while high-quality development is defined by industrial development at the medium level.

Green production methods must be used throughout the entire process in order to achieve the goal [19]. Taking pig fattening and farming as an example, from the perspective of the material flow process, the farming process involves significant links, such as the feeding of piglets and feed, disease prevention and control, feeding supervision and manure treatment. The corresponding green production behaviors should include reducing feed, scientifically preventing and controlling diseases, standardizing the process management and timely disposing of manure to promote resource conservation, product safety, environmental friendliness and efficient output. Based on this process, this paper proposes that the green production behaviors of pig farming include the following four specific behaviors: The first is the rationalization of feeding. As feed and piglet costs account for more than 80% of pig farming costs [20], a proper feed–nutrition ratio and an improved variety selection can help to lower feed costs and realize resource saving and output efficiency. The second is scientific epidemic control. The occurrence and prevalence of livestock and poultry epidemics have significantly reduced the slaughter rate and the efficiency of output. Therefore, the scientific and effective prevention and control of epidemics is a crucial step to realize the transformation, upgrading and sustainable development of the livestock industry [21]. At the same time, scientific disease control is also an important means to reduce drug residues and ensure product safety. The third is standardized management. A clean farming environment contributes to the healthy growth of pigs [22] and is the result of the routine standardized management of pig farms. Standardized management helps to reduce waste and is also an effective guarantee of product safety and output efficiency. The fourth is manure recycling. The solution to the challenging issues in rural environmental governance and pollution control lies in the recycling of waste from livestock and poultry farms [23]. It is a crucial behavior to achieve environmental advantages and, as a result, support from external stakeholders. Table 1 shows the four green production practices mentioned above and their specific performances.

### 2.2. Theoretical Analysis

If the marginal revenue of implementing a new technology or production method is higher than the marginal cost, the implementation of such a method is the best option [24]. A new technology or method should be implemented only if the net income is higher than the income before implementation [25]. Based on the theory of the Rational Peasant, this paper argues that the first consideration for small- and medium-sized pig farmers looking to implement green production behaviors is relatively certain economic returns; the decision is made after referring to the change of net incomes before and after implementation. Thus, the conditions for implementing green production behaviors can be set as:

(1) $$pg\left(n\right)e\left(z\right)-\left(w+c\right)n\geq p_{0}f\left(n\right)-cn$$

In the Formula (1), *p* is the price of the product after the green production behavior is implemented; *g* (·) is the production function after the implementation; *e* (*z*) represents the subjective risk function determined by *z*; *e* (*z*)∈ [0, 1], and is the combination of influencing factors of the green production behaviors of pig farmers, such as the external environment characteristics and the pig farmers’ own endowment characteristics; *w* is the incremental cost per unit for implementing the green production behaviors; *c* is the unit cost before the implementation; *n* is the farming scale; *p*_0_ is the price of the product before implementation; and *f* (·) is the production function when the green production behaviors are not implemented. After the implementation of green production behaviors, the pork quality is higher than before, and its price, too, that is, *p* ≥ *p*_0_.

Considering that price, cost and production function are determined by the objective economic and technological conditions, the subjective risk function *e (z)* is the major factor that determines whether farmers adopt green farming practices. Formula (1) can be transformed to determine the prerequisites for applying green production behavior:

(2) $$e\left(z\right)\geq \frac{p_{0}f\left(n\right)+wn}{pg\left(n\right)}$$

In the Formula (2), each variable on the right is greater than 0 and is determined by objective economic and technological factors that can be considered as a reference point (critical value) for making decisions on green production behaviors, and will be set as an unknown constant *K*_0_. Then, the Formula (2) is converted into *e* (*z*) ≥ *K*_0_. Let *Y* be the decision variable for implementing green production behaviors; with the two endpoints “yes” and “no”, it is a binary decision issue. If *e* (*z*) ≥ *K*_0_, then *Y* = 1, which means that the pig farmer implements green production behaviors; otherwise, *Y* = 0, indicating no implementation of green production behaviors. Following the classical hypothesis, *z* is considered as a linear function of a series of factors that contribute to the green production behaviors of pig farmers:

(3) $$z=\alpha +\sum_{i=1}^{m}\beta_{i}x_{i}+\mu$$

In Formula (3), *α* and *β* are parameters to be estimated, *x_i_* is a series of conditional factors of item *i* of pig farmers and *μ* is a random variable subject to extreme value distribution. In the empirical study, the relationship between various characteristics and *z* can be found out by analyzing *x_i_* in order to analyze *e* (*z)*, which contributes to the green production behaviors of farmers.

### 2.3. Research Hypotheses

What factors contribute to the green production behaviors of pig farmers? Implementing green production behaviors is a typical uncertain decision, which can be explained by the Prospect Theory in behavioral economics. The main focus of the theory, developed by Daniel Kahneman and Amos Tversky, is on how behavioral agents choose behaviors while taking into account expected returns in various scenarios. The theory’s key components include certain effect, reflection effect, loss aversion and reference dependence [26]. The “certain effect” means that the decision-maker chooses the certain return over the uncertain return. The “reflection effect” represents the decision-maker’s choosing of the uncertain return over the certain loss; the “loss aversion” refers to the asymmetry of sensitivity of the decision-maker to loss and gain. The pain of loss is much greater than the pleasure of gain. “Reference dependence” means that decision-makers’ judgment on gain and loss comes from comparison. Most people tend to judge gain and loss in accordance with reference points, and they pick “gain” when they receive more than the reference points [27].

According to the Prospect Theory, farmers first consider if they can get a “certain return” before deciding whether to use green farming practices [28]. According to the Rational Peasant theory, farmers are willing to take such measures when there are specific economic benefits. The second aspect they will take into account is loss aversion, or the aversion of the severe agony of loss. Due to strict environmental regulations and other rules, farmers may face serious penalties once they fail to meet the environmental protection standards. In order to avoid the pain caused by penalties, farmers may choose to implement green production behaviors to obtain environmental benefits [29]. The third factor they will consider is the reflection effect. Farmers may experience “losses” in the early stages of transformation since costs are higher than gains, but the social benefits (e.g., high-quality and safe pork) may bring additional profits to farmers. At this time, farmers will also choose to implement green production behaviors [30]. Finally, regarding the reference dependence, farmers will compare the gains and losses before and after the implementation of green production behaviors. If the gains are greater than the losses, they will choose to implement the behavior, but the long-term or growth benefits may be considered [6]. Extensive study has already been conducted on the influence of farmers’ cognition of return on their green production behaviors, with a focus on the role of economic benefit [31,32], environmental benefit [10] and social benefit [15]. Scholars agree that the higher the farmers’ cognition of the benefits of green production, the more motivated they are to participate in green production. To sum up, farmers’ cognition of return is a necessary condition and an important influencing factor in determining the behavior of green production, including the cognition of economic effect, social effect, environmental effect and growth effect. For this purpose, this paper proposes Hypothesis 1:

**Hypothesis 1.**  *Cognition of return has a significant impact on the green production behaviors of small- and medium-sized pig farmers, and the bigger the cognition of return, the more likely the behavior will be performed.*

Generally speaking, the more pressure an individual feels to act from others or groups (the public, the government, consumers, etc.) in action, the more likely it is that their intentions will be carried out [33]. In terms of pig farming, local governments shoulder the responsibility of coordinating social, economic and ecological development. They also direct or impose restrictions on the production and operation activities of farmers by enacting rules and regulations. Local residents demand that farming production and operation activities do not bring adverse effects on the environment and image of the community. Consumers may have high requirements for product quality and may supervise the whole process of product formation through a traceability system. Pig farmers must adapt their conduct quickly to the various stockholders’ needs in order to promote long-term development. Existing studies prove that national environmental regulation has an important role in encouraging farmers to engage in green production behaviors; the stricter the regulation, the more likely it is that farmers will undergo a green transformation [7]. In addition, external monitoring and consumer demand are also important factors of farmers’ transition to sustainable farming, which can effectively encourage farmers to implement green production behaviors [34,35,36]. Therefore, external pressure is also an important factor for farmers to implement green production behaviors [37]. This leads to Hypothesis 2:

**Hypothesis 2.**  *The degree of external pressure has a substantial impact on the green production practices used by pig farmers, and the more pressure there is, the more likely it is that the practices will be used.*

According to the Resource-based Theory, the occurrence of behaviors is not only affected by behavioral intention, but also restricted by objective control conditions such as the ability to implement behaviors and some resources [38]. Farmers need technology, facilities, land and other resources to apply green production practices, such as necessary agricultural land to store manure for disposal and equipment to deal with manure. Additionally, green production behavior is more likely to be performed by pig farmers with stronger resource capacities and more favorable environmental conditions [39]. Some scholars have confirmed that resource endowments, including technology, facilities and natural resources, can facilitate the transformation of farmers’ green production intentions into green production behaviors and have a positive impact on farmers’ green production behaviors [40,41]. This leads to Hypothesis 3:

**Hypothesis 3.**  *Pig farmers are more likely to adopt sustainable production practices the more resources they have at their disposal.*

Farmers’ individual traits establish their level of skill, shape their judgment cognition on their green production behavior and ultimately affect how they choose to act [13]. In general, male farmers are more aware of the effects of green production behaviors on disease prevention and input saving in pig farming, so they may pay more attention to the implementation of green production behaviors. Young farmers have a stronger sense of modern farming and are more likely to adopt green production behaviors [42]. Farmers with a higher education level have a deeper understanding of the comprehensive effects of green production and a stronger sense of social responsibility, so they are more willing to implement green production behavior [43]. Farmers with expertise in animal husbandry, economics and management are more willing to carry out standardized farming and are more likely to implement green production methods [44]. Thus, Hypothesis 4 is proposed:

**Hypothesis 4.**  *Male, younger, more educated and more specialized pig farmers are more likely to implement green production practices.*

Farmers’ operational state is a key determinant of their operational capacity and of their adoption of sustainable production practices [13]. Generally speaking, the longer farmers are engaged in the farming industry, the more farming experience they have accumulated, the better they are at timing green production behaviors, and the more helpful it is to do so [45]. Existing research demonstrates that adopting green production practices is more likely to occur on a large-scale farm and is also positively correlated with the operating profit from the previous year [46]. Thus, Hypothesis 5 is proposed:

**Hypothesis 5.**  *Green production practices are more likely to be used by farmers that have been in operation for a long time, operate at a bigger size and have had better operating results the previous year.*

**Figure 1:**
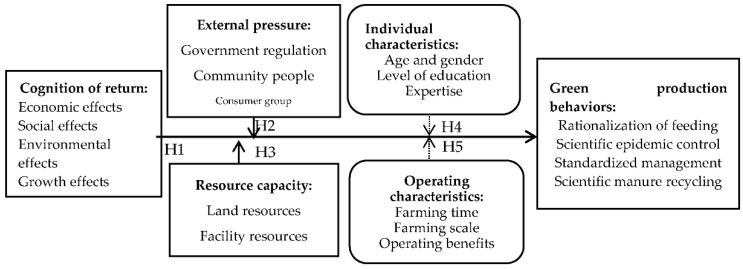
Influencing Factors of the Green Production Behaviors of Farmers.

According to the above analysis, the factors that contribute to the green production behaviors of pig farmers include cognition of return, external pressure, resource capacity, individual characteristics and operating characteristics, as shown in Figure 1.

## 3. Estimation Models, Variables Measurement and Data Sources

### 3.1. Estimation Model

#### 3.1.1. Logit Model

Since the explained variables are discrete binary selection variables, this paper uses the binary Logit model to estimate the influencing factors of the green production behaviors of farmers.

(4) $$P=F(y=1/x_{i})=\frac{1}{1+e^{-y}}$$

(5) $$y=\ln(\frac{P}{1-P})$$

where *P* represents the probability that farmers implement green production behaviors and *y* is the explained variable—that is, whether pig farmers adopt green production behavior; *y* = 1 means the farmers have implemented green production behavior, and *y* = 0 means the farmers have not implemented green production behavior. The variable *x_i_* (*i* = 1, 2… *n*) is the explanatory variable—that is, the factors that may contribute to the green production behavior of pig farmers. The explained variable *y* is a linear combination of the explanatory variables *x_i_* (*i* = 1, 2… *n*), expressed as:

(6) $$y=\alpha +\sum_{i=1}^{m}\beta_{i}x_{i}+\varepsilon$$

where α represents the constant term, β is the coefficient to be estimated for the explanatory variable, *xi* (*i* = 1, 2… *n*) is the explanatory variable and ε is the random error term.

#### 3.1.2. ISM Analysis Method

A number of factors work together to influence the occurrence of green production behaviors, rather than a single one. The way a behavior develops is a result of the interaction of several elements. In order to analyze the interaction of different factors, this paper chooses the ISM (Interpretive Structural Modeling) method to analyze the correlation and multi-step structure among the influencing factors of the green production behaviors of pig farmers.

The ISM analysis method was proposed by Professor John N. Warfield in the United States for analyzing the structure of complex social and economic systems. The basic principle is that by determining the various factors that affect the system and their mutual relations using the correlation matrix principle in graph theory and computer technology, the information about the factors and their mutual relations is processed so as to clarify the relevance and level of the factors and discover the key factors and their internal relations [47].

ISM is usually used to study the internal structure and hierarchy of the system, decompose the complex system into several subsystems (elements), and analyze the correlation and hierarchical structure among the subsystems (elements). The advantage of this model is that it can structure the system by dividing the decision criteria into different levels according to its driving and dependent capabilities [48]. Therefore, ISM is used in this paper to analyze the correlation and hierarchy among various factors that contribute to farmers’ green production behaviors.

The main analysis steps of the ISM analysis method are as follows: determine the key influencing factors, construct the adjacency matrix according to the relation graph, obtain the accessibility matrix, decompose the accessibility matrix and establish the structure model.

In this paper, *S*_0_ is used to represent the green production behavior of pig farmers. If there are *k* influencing factors of the green production behavior analyzed by the Logit model, *S_i_* (*i* = 1, 2,…, *k*) is used to represent the green production behavior. According to experts’ judgment on the logical relationship among the factors, the logical relationship graph is constructed, and then the adjacency matrix of the factors is determined.

(7) $$a_{ij}=\left\{\begin{array}{c} 1,\;S_{i}\;has\;an\;effect\;on\;S_{j}\\ 0,\;S_{i}\;has\;no\;effect\;on\;S_{j} \end{array}\right.\;i=0,\;1,\;\ldots \ldots ,\;k;\;j=0,\;1,\;\ldots \ldots ,\;k$$

The matrix A composed of $a_{ij}\;$is the adjacency matrix, and the formula for converting the adjacency matrix into the accessibility matrix is as follows:

(8) $$M={\left(A+I\right)}^{\lambda +1}={\left(A+I\right)}^{\lambda }\neq {\left(A+I\right)}^{\lambda -1}\neq \cdots \neq {\left(A+I\right)}^{2}\neq \left(A+I\right)$$

where I is the unit matrix, 2 ≤ *λ* ≤ *k*, and the above matrix operation follows the Boolean operation rule. The highest-level factor is determined as follows:

(9) $$L_{1}=\left\{S_{i}|P\left(S_{i}\right)\cap Q\left(S_{i}\right)=P\left(S_{i}\right);i=0,1,\ldots \ldots ,k\right\}$$

where, $P\left(S_{i}\right)$ is the accessible set, including all factors that can be directly or indirectly reached from the $S_{i}$ in the accessibility matrix; $Q\left(S_{i}\right)$ is the antecedent set, including all factors that can directly or indirectly reach $S_{i}$ in the accessibility matrix, and $P\left(S_{i}\right)$ and $Q\left(S_{i}\right)$ are expressed as:

(10) $$P\left(S_{i}\right)=\left\{S_{j}|m_{ij}=1\right\},\;Q\left(S_{i}\right)=\left\{S_{j}|m_{ji}=1\right\}$$

where $m_{ij}$ and $m_{ji}$ are the elements of the accessibility matrix M. The other levels of factors are determined as follows: delete the rows and columns corresponding to the L_1_ factors from the accessibility matrix M to obtain the accessibility matrix M′; perform the operations of Formulas (8) and (9) on the accessibility matrix M′ again to obtain the L_2_ factors; delete the rows and columns corresponding to the L_2_ factors from the matrix M′ to obtain the matrix M″; operate the matrix M″ in the same way to obtain the L_3_ factors, and so on, and finally obtain the factors at all levels. According to the relationship between each level, the factors of each level are connected to obtain the correlation chromatography relationship among the influencing factors of the pig farmers’ green production behavior.

### 3.2. Selection of Variables

According to Figure 1, four green production behaviors are selected as the explained variables, and sixteen variables in five types are selected as the explanatory variables.

#### 3.2.1. Explained Variables

The green production behaviors of pig farmers include rationalization of feeding, scientific epidemic control, standardized management and manure recycling. The specific performances of the four types of behaviors are shown in Table 1. This paper holds that as long as one answer is Yes in each specific performance, the value is 1, and if all answers are No, the value is 0.

#### 3.2.2. Explanatory Variables

According to the above hypotheses, 16 explanatory variables in 5 categories are selected to analyze the influencing factors and their interaction mechanism of the green production behaviors of pig farmers.

The cognition of return includes farmers’ cognition of expected economic, environmental, social and growth benefits once green production behaviors are implemented. Economic benefit refers to the increase of income that pig farmers expect if they are to implement green production behaviors; social benefit refers to the expected effect of pig farmers’ green production behaviors on the improvement of product quality; environmental benefit refers to the expected environmental effect of the green production behaviors; the growth benefit refers to the growth of pig farmers in technology, management and other aspects by implementing green production behaviors. In accordance with the cognition degree of farmers, the cognitive indicators are divided into 5 levels, from completely disagree to completely agree, which are represented by 1~5, respectively.

External pressure includes government regulation, consumer demand and social norms. Government regulation means that the government urges the green development of farming industry by issuing policies and opinions, measured by the degree of government’s advocacy for green production; consumption demand means the demand of consumers for improved product quality, measured by the requirements for green products; social norms refer to the supervision of various social groups, especially local residents, to promote the green production of the farming industry, measured by the social supervision of green production. In accordance with the cognition degree of farmers, the external pressure indicators are divided into 5 levels, from completely disagree to completely agree, which are represented by 1~5 respectively.

The resource capacity includes the resource endowment and farming capacity of the pig farmers to carry out green production. Resource endowment refers to the natural and social resources required for green production, which is measured by whether there is arable or forest land around the farm. Yes is recorded as 1. No is recorded as 0. Farming capacity refers to the technology and equipment possessed by the farmers to carry out green production, measured by the configuration of the automation technology and equipment in the farm. The condition basis is divided into five levels. The more conditions you have, the stronger your ability is, represented by 1 to 5.

The control variables are the individual characteristics and operating characteristics of the farmers. Individual characteristics include sex, age, level of education and specialty of farm owners, while operating characteristics include farming time, breeding stock at the end of the previous year and farming profit at the end of the previous year. The details of each variable are shown in Table 2.

### 3.3. Data Source and Sample Statistics

The data used in this paper were obtained through a questionnaire survey conducted in 115 counties (county-level cities and districts) in Henan Province from January to February of 2021. Henan Province is one of the largest pig-farming provinces in China, and there were 43.9 million pigs raised in 2021 accounting for 9.8 percent of the total; there were about 410,000 farmers, of which small- and medium-sized farmers (including retail) accounted for 96%. The questionnaire mainly included the individual characteristics of the farmers, household characteristics, farmers’ green production behaviors and willingness, cognition of return, external pressure and resource capacity, etc.

The survey adopted the method of door-to-door investigation. Twenty-five postgraduate students were recruited as investigators from Henan Agricultural University. In order to avoid subjective errors, the investigators were trained intensively before the formal investigation. Then, they conducted one-to-one interviews with farm owners to finish the questionnaires. A total of 800 questionnaires were distributed to pig farmers, and 789 ones were recovered. After excluding invalid questionnaires, 763 valid questionnaires were obtained, with an effective rate of 95.3%. Among them, there were 747 small- and medium-sized farms, including 169 individual pig farmers (22.1%), 408 small-sized farms (53.5%), and 170 medium-sized farms (22.3%). The sample characteristics are presented in Table 3.

As can be seen from Table 3, the pig farmers are mainly male, accounting for 91.4 percent; the farmers aged 40–59 years account for 75.3 percent; full-time farming and semi-farming account for 95.1 percent of the total, with a high degree of specialization. In addition, most of the farmers have a junior or senior high school degree, accounting for 78.4% of the total sample. The education level of the medium-sized farmers is higher than that of the individual and small-sized farmers. The samples are typical as they are basically consistent with the current situation of pig farming in rural areas.

To be clear, full-time farming means that pig farming is the only source of income for farmers; semi-farming means that farm owners both plant crops and raise pigs, with pig farming providing more than half of their income; part-time farming means that farm owners are engaged in both pig farming and non-agricultural activities, with pig farming providing less than half of their income.

## 4. Estimation Results and Discussion

### 4.1. Descriptive Statistics

A descriptive statistical analysis of the variables was carried out, and the results are shown in Table 2. According to Table 2, 70%, 70%, 76% and 77% of pig farmers implemented green production behaviors of rationalization of feeding, scientific of epidemic control, standardized management and manure recycling, respectively. It shows that there were a large proportion of farmers who implemented green production behaviors. The average values of economic benefit, social benefit, environmental benefit and growth benefit were 3.69, 3.67, 3.64 and 3.66, respectively, which were generally higher than the average level of 3. In terms of external pressure factors, the average values of government regulation, consumption demand and social norms were 3.62, 3.66 and 3.63, respectively, indicating that the requirements of the government, society and organizations on green production behaviors considered by farmers are close to a relatively higher level. In the factor of resource capacity, the average value of resource endowment was 0.59—that is, only 59% of farmers had arable or forest land and could carry out green farming by combining planting and farming such as manure storage for disposal; the average value of farming capacity was 3.59, indicating that the technical equipment level was above the medium level.

In terms of control variables, 91% of farm owners were male; the average age of the farm owners was 49.34, and the average age of the workers was moderate; the average level of education was 1.97, and most of the farmers had received education in junior middle school or below; 43% of the farmers had received systematic learning or training in animal husbandry and operation management; and the average farming time for the farmers was 12.92 years. In 2020, the average number of pigs in small- and medium-sized farms was 102.01, and the average profit was CNY 1,151,800, with a large standard deviation and large fluctuations among all pig farms.

### 4.2. Analysis of Influencing Factors of Green Production Behaviors

In this paper, Stata14.0 was used for the regression analysis of Equation (6), and the estimation results are shown in Table 4. Model 1 is the result of the regression of all 16 variables of different green production behaviors through the Logit model, and Model 2 is the result of regression again for the variables that are significant at the 10% level selected by Model 1.

As can be seen from Table 4, the R^2^ values of Model 2 in the regression models of feeding rationalization, scientific epidemic control, standardized management and manure recycling were 0.581, 0.859, 0.664 and 0.683, respectively, and the significance level was 0.000, indicating that the data were well fitted and could be used for analysis. First of all, from the regression results of Model 2, we can see that 10 factors were statistically significant to the rationalization of feeding: economic benefit, social benefit, growth benefit, social norm, resource endowment, farming ability, level of education, specialty, breeding stock and profit. The cognition of environmental benefits, government regulations and consumption demands were not statistically significant; the age, sex, and working time of the owners failed to pass the significance test. Secondly, 11 factors were statistically significant to the scientific epidemic control: cognition of economic benefit, social benefit, environmental benefit, growth benefit, government regulation, organizational involvement, social norm, farming ability, level of education, breeding stock and profit; the resource endowment failed to pass the test. The age, sex, working time and expertise in individual and operating characteristics failed to pass the significance test. Thirdly, nine factors were statistically significant to the standardized management of pig farmers: cognition of economic benefit, social benefit, environmental benefit, growth benefit, government regulation, organizational involvement, social norm, farming ability and level of education. The resource endowment failed to pass the test. Among the individual and operating characteristics, only the level of education was positively correlated with the standardized management. Finally, nine factors were statistically significant to manure recycling of pig farmers: cognition of economic benefit, social benefit, environmental benefit, growth benefit, government regulation, social norm, farming ability, level of education and profit; in the external pressure, consumer demand and arable or forest land resources failed to pass the test. In addition, among the individual and operating characteristics, the age, sex, working time and the breeding stock of the previous year failed to pass the test.

Factors related to the above four types of behaviors included cognitive factors such as economic benefits, social benefits and growth benefits, social norms, farming capacity and years of education. Factors that were not related included age, sex, working time of the farm owners, and so on. This indicates that the green production behavior of farmers is mainly driven by the internal economic, social and growth benefits and by the external supervision of the community, and supported by necessary technical equipment. It is highly correlated with the years of education of the pig farmers but has nothing to do with their age, sex and working time.

### 4.3. Explanatory Structure of Influencing Factors of Green Production Behaviors

The above-mentioned factors of green production behaviors interact and are independent of each other, but the above-mentioned regression model cannot determine the relationship between the influencing factors. For this reason, this section uses the ISM analysis method to explore the relationship between the factors.

**Figure 2:**
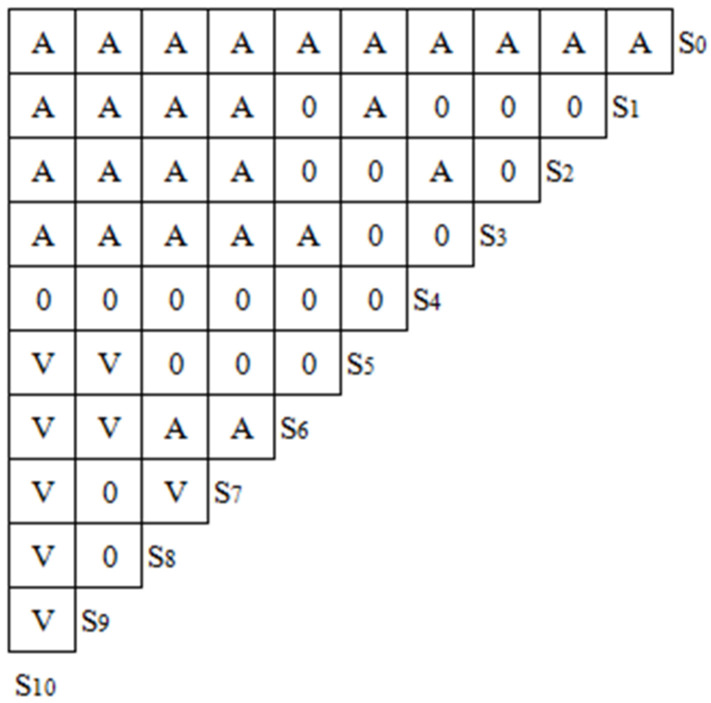
Logical Relationship Among Influencing Factors of Feeding Rationalization.

In the light of the research result of the influencing factors of the feeding rationalization, the influencing factors are listed. In this paper, S_1_, S_2_, S_3_, S_4_, S_5_, S_6_, S_7_, S_8_, S_9_ and S_10_ are respectively used to represent cognition of economic benefit, social benefit, growth benefit, social norm, resource endowment, farming ability, level of education, specialty, breeding stock and profit. On the basis of analysis, discussion and consultation with experts and scholars, the logic diagram of the factors is made. As shown in Figure 2, A indicates that the column factors have a direct or indirect effect on the row factors, and V indicates that the row factors have a direct or indirect effect on the column factors. For example, all the factors of S_1_–S_9_ have a direct or indirect influence on S_0_ of farmers’ green production behaviors; S_5_–S_9_ of resource endowment, farming ability, level of education, specialty, breeding stock have a direct or indirect influence on the S_10_ of profit.

The adjacency matrix B is determined according to Figure 2 and Formula (7), and the accessibility matrix M is obtained according to Formula (8) using Matlab7.11 software.

(11) $$B=\begin{array}{c} S_{0}\\ S_{1}\\ S_{2}\\ S_{3}\\ S_{4}\\ S_{5}\\ S_{6}\\ S_{7}\\ S_{8}\\ S_{9}\\ S_{10} \end{array}\left|\begin{array}{ccccccccccc} 0 & 0 & 0 & 0 & 0 & 0 & 0 & 0 & 0 & 0 & 0\\ 1 & 0 & 0 & 0 & 0 & 0 & 0 & 0 & 0 & 0 & 0\\ 1 & 0 & 0 & 0 & 0 & 0 & 0 & 0 & 0 & 0 & 0\\ 1 & 0 & 0 & 0 & 0 & 0 & 0 & 0 & 0 & 0 & 0\\ 1 & 0 & 1 & 0 & 1 & 0 & 0 & 0 & 0 & 0 & 0\\ 1 & 1 & 0 & 0 & 0 & 0 & 0 & 0 & 0 & 1 & 1\\ 1 & 0 & 0 & 1 & 0 & 0 & 0 & 0 & 0 & 1 & 1\\ 1 & 1 & 1 & 1 & 0 & 0 & 1 & 0 & 1 & 0 & 1\\ 1 & 1 & 1 & 1 & 0 & 0 & 1 & 0 & 0 & 0 & 1\\ 1 & 1 & 1 & 1 & 0 & 0 & 0 & 0 & 0 & 0 & 1\\ 1 & 1 & 1 & 1 & 0 & 0 & 0 & 0 & 0 & 0 & 0 \end{array}\right|$$

(12) $$M=\begin{array}{c} S_{0}\\ S_{1}\\ S_{2}\\ S_{3}\\ S_{4}\\ S_{5}\\ S_{6}\\ S_{7}\\ S_{8}\\ S_{9}\\ S_{10} \end{array}\left|\begin{array}{ccccccccccc} 1 & 0 & 0 & 0 & 0 & 0 & 0 & 0 & 0 & 0 & 0\\ 1 & 1 & 0 & 0 & 0 & 0 & 0 & 0 & 0 & 0 & 0\\ 1 & 0 & 1 & 0 & 0 & 0 & 0 & 0 & 0 & 0 & 0\\ 1 & 0 & 0 & 1 & 0 & 0 & 0 & 0 & 0 & 0 & 0\\ 1 & 0 & 1 & 0 & 1 & 0 & 0 & 0 & 0 & 0 & 0\\ 1 & 1 & 1 & 1 & 0 & 1 & 1 & 0 & 1 & 1 & 1\\ 1 & 1 & 1 & 1 & 0 & 1 & 1 & 0 & 1 & 1 & 1\\ 1 & 1 & 1 & 1 & 0 & 1 & 1 & 1 & 1 & 1 & 1\\ 1 & 1 & 1 & 1 & 0 & 1 & 1 & 0 & 1 & 1 & 1\\ 1 & 1 & 1 & 1 & 0 & 1 & 0 & 0 & 1 & 1 & 1\\ 1 & 1 & 1 & 1 & 0 & 1 & 0 & 0 & 1 & 1 & 1 \end{array}\right|$$

On the basis of the accessibility matrix, L_1_ = {S_0_} is obtained by Equations (9) and (10). Then, the other levels of factors are obtained in turn according to the operation method, L_2_ = {S_1_, S_2_, S_3_}, L_3_ = {S_4_, S_5_, S_6_, S_8_, S_9_, S_10_}, L_4_ = {S_7_}. The accessibility matrix M is reordered according to the obtained L_1_, L_2_, L_3_ and L_4_ to obtain the accessibility matrix R.

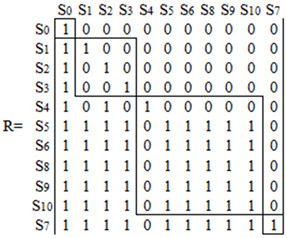
(13)


**Figure 3:**
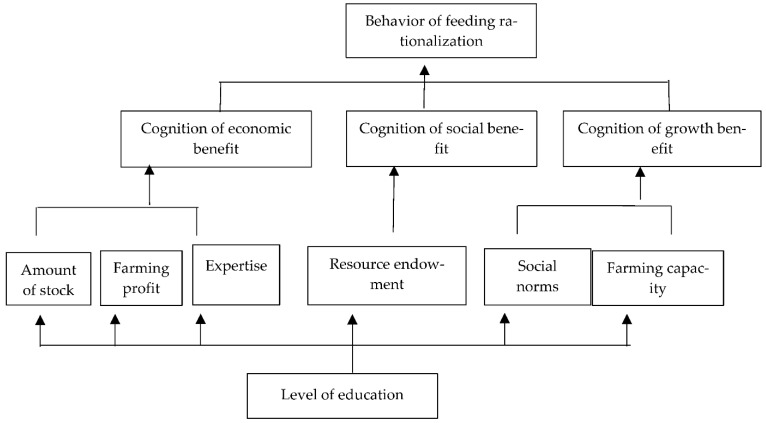
Correlation and Hierarchical Structure Among Influencing Factors of Feeding Rationalization.

According to the accessibility matrix R, S_0_ is in the first layer, S_1_, S_2_ and S_3_ are in the second layer; S_4_, S_5_, S_6_, S_8_, S_9_ and S_10_ are in the third layer; and S_7_ is in the fourth layer. The influencing factors of the same level and adjacent levels are linked to each other with directed edges, and the correlation and hierarchical structure among the influencing factors of pig farmers’ feeding rationalization are obtained, as shown in Figure 3.

**Figure 4:**
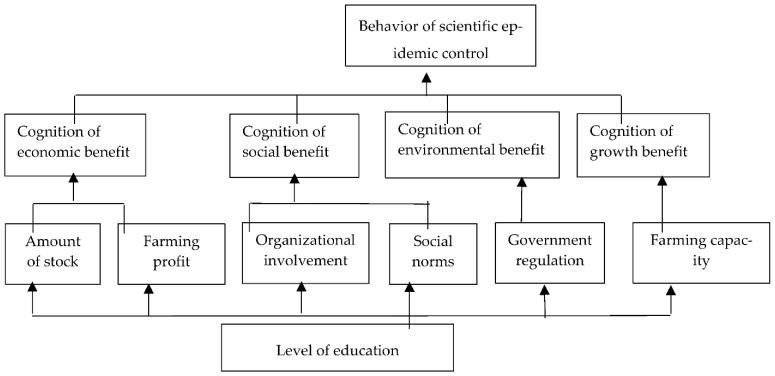
Correlation and Hierarchical Structure Among Influencing Factors of Scientific Epidemic Control.

**Figure 5:**
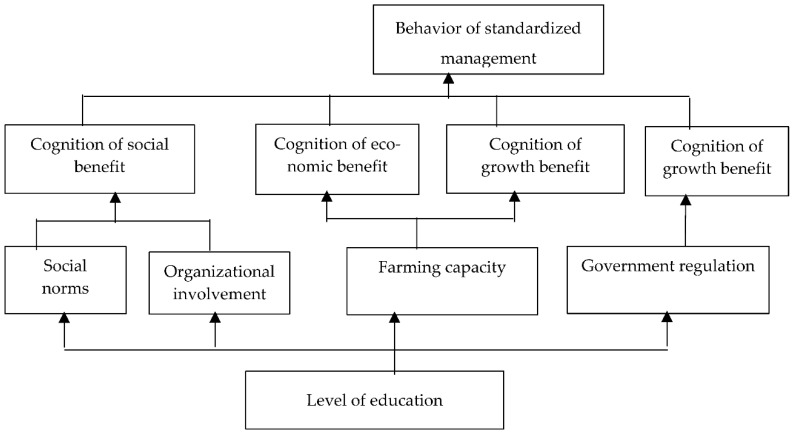
Correlation and Hierarchical Structure Among Influencing Factors of Standardized Management.

**Figure 6:**
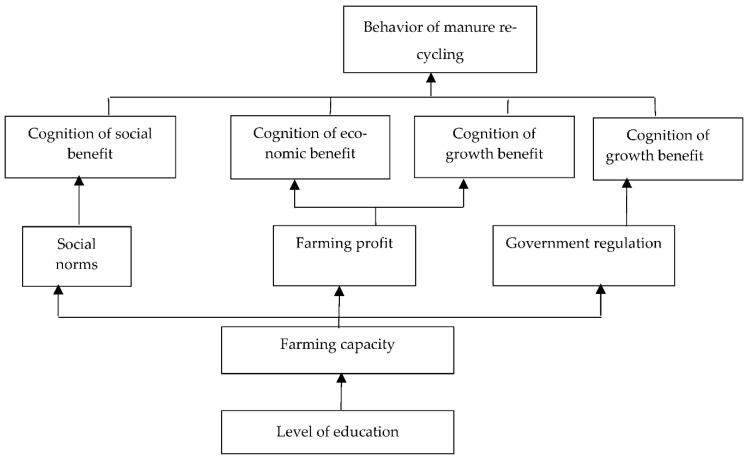
Correlation and Hierarchical Structure Among Influencing Factors of Manure Recycling.

In the same method and step, the correlation and hierarchical structure among the influencing factors of the scientific epidemic control, standardized management and manure recycling can be obtained. The results of the analysis are presented in Figure 4, Figure 5 and Figure 6.

Taking the structural explanation of the influencing factors of feeding rationalization as an example, as can be seen from Figure 3, the result is as follows: among the influencing factors of the behavior of feeding rationalization of the pig farmers, cognition of economic benefit, social benefit, growth benefit are surface-level direct factors. Social norms, resource endowments, farming capacity, breeding stock, farming profits and specialties are indirect factors, and education level is the underlying factor.

Analysis of direct factors: The regression coefficients of cognition of economic benefit, social benefit and growth benefit were 0.865, 1.207 and 0.880, respectively, and the *p* value was 0.000, which was significant at the 1% level. That is to say, the three factors all had a very significant positive influence on the feeding rationalization of pig farmers, which is consistent with the expected result. Cognitive psychology shows that cognition affects behavior, and behavior is the external performance of cognition. Farmers who have a good cognition of the expected benefits of green production behavior are more likely to put their intention into action. Farmers with green farming technology and equipment help facilitate the rationalization of feeding and the long-term stable development of farms, supply high-quality pork to the market, and improve the expected economic, social and development benefits of farming. Cognition affects intention, and intention determines behavior. Farmers are more inclined to use green farming technologies and have a better chance of doing so if they have a clearer understanding of the rationalization of feeding.

Analysis of indirect factors: The regression coefficient of social norms was 1.414, and the *p*-value was 0.000—that is, social norms had a positive influence on the rationalization of feeding at the significance level of 1%, which is consistent with the expected result. Through daily supervision, local residents exert pressure on pig farming activities, urge pig farmers to improve farming methods and develop green farming for the purpose of environmental protection and energy conservation. Under the social norms, farmers can obtain a series of social benefits such as enterprise credit, social funding and public support by implementing the rationalization of feeding behavior. When farmers have a clear understanding of this, they are more likely to adopt this behavior, and the effect of social norms on the cognition of social benefits has an impact on the rationalization of feeding behavior.

The regressive coefficients of resource endowment and farming capacity were 1.041 and 0.390, respectively, and the *p* values were 0.001 and 0.024, respectively, indicating that resource endowment and farming capacity had a significant positive effect on the feeding rationalization of pig farmers, which is consistent with the expected results. According to the theory of planned behavior, the production of behavior is influenced not only by will but also by ability, which can guarantee the production of behavior. The ability includes resources and conditions. In order for pig farmers to rationalize their feeding activities, they should base their efforts on natural resources such as land, water, energy, and also need assistance from basic conditions such as human resources, technology and automatic facilities. Sufficient resources, talents and facilities are guarantees of the implementation of behaviors and also promote the long-term development of the farms. Only when sufficient conditions are available can the farmers have expectations for future development and generate awareness of the growth benefits of green production behaviors. Therefore, resource endowment and farming ability play an important role in the growth and cognition of return, which urges the farmers to implement the rationalization of feeding.

The regression coefficients of expertise and farming profit were 1.390 and 0.040, respectively, and the *p*-value score ratio was 0.000 and 0.056, respectively, indicating that expertise and farming profit had a significant positive effect on the rationalization of feeding, which is consistent with the expectation. Accepting professional learning and mastering professional skills are prerequisites for pig farming activities. Through professional learning, it is possible to accurately capture industry information and reasonably arrange the farming process so as to maximize the use of natural, social and human resources and ensure low cost and high efficiency. Farming profit is the most important basis for farmers to judge the development prospect and direction of the industry. A higher profit leads to a higher economic profit expectation of feeding rationalization and a greater possibility of implementation. The regression coefficient of breeding stock was −0.004 and the *p* value was 0.000, indicating that the breeding stock had a negative influence on the rationalization of feeding, which is inconsistent with the expected results. The reason may be that it is more cost-effective to use a uniform feed when stocks are large. The higher the expected economic effect, the more likely it is for the farmers to adopt the rationalization of feeding. The farming profit, specialty and stock had a direct impact on the estimation of the economic benefit of the pig farmers. They all acted on the cognition of economic benefit and their rationalization of feeding.

Analysis of the fundamental factor: The regression coefficient of education level was 0.866 and the *p* value was 0.000, indicating that the education level had a very significant positive effect on the feeding rationalization of pig farmers, which is consistent with the expected result. The higher the level of education, the stronger the individual capacity of farmers; decision-making and implementation capacity also improved. With the improvement of individual education level, they can accurately capture the development direction of the pig farming industry advocated by the government, society and organizations. With the enhanced information collection ability, they can effectively use various preferential measures and subsidy policies to realize the green transformation at a low cost. The farming capacity will be enhanced with a higher education level; the farming quality and efficiency will be improved by mastering more farming skills through professional learning, so as to increase the farming income, expand the farming scale and realize a virtuous cycle. A high education level creates a good condition for the implementation of rationalization of feeding. The effect of behavioral cognition and willingness is manifested as a better understanding of the benefits of rationalization of feeding, a stronger willingness to implement the behavior, and an increased possibility of putting the willingness into action.

In addition, the cognition of environmental benefits, government regulation, organizational involvement, farmer’s gender, age and farming time had no significant influence on the feeding rationalization. The possible reason is that feeding rationalization acts directly on economic, social and long-term development, and farmers have little understanding of the environmental effects, so the cognitive effects of environmental benefits are limited. The influence of government regulation and organizational involvement on the rationalization behavior is not as direct as the market demand, which is also limited. Since feeding rationalization is required by social norms and is related to the survival and development of pig farms, the gender, age of the owner and the farming time of the farm have little influence on the social needs and benefits.

In conclusion, the above 10 factors are independent, interrelated and constitute a system of influencing factors on the feeding rationalization of pig farmers. The specific action path and transmission mechanism of the influencing factors are education level → social norm, resource endowment, farming ability, specialty, farming profit, breeding stock → cognition of economic benefits and social benefits, growth benefits → rationalization of feeding.

**Figure 7:**
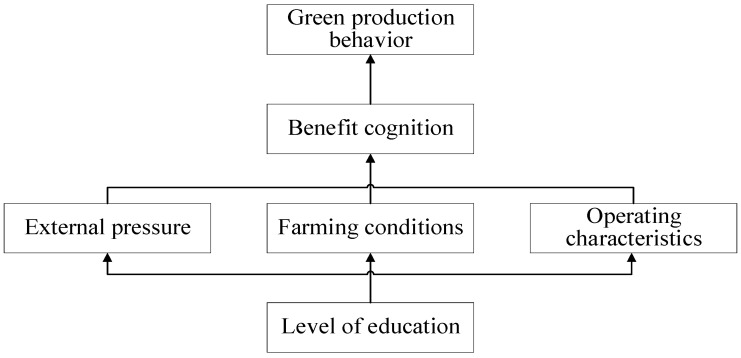
Correlation and Hierarchical Structure Among Influencing Factors of Green Production Behavior.

Referring to Figure 4, Figure 5 and Figure 6, the structural explaination of the influencing factors of the other green production behavior, such as scientific epidemic control, standardized management and manure recycling, is the same as the one of the rationalization of feeding behavior, which is not described here. The conclusion of the structural analysis of the four kinds of green production behaviors can be obtained as follows: the cognition of return status of green production behaviors is the direct factor; the external pressure, farming conditions and operating characteristics are indirect factors; and the education level is the underlying factor. That is, based on the education level, the improvement of pig farmers’ own ability, the increase of external pressure, the improvement of farming conditions and other factors all act on farmers’ cognition of the expected benefits of green farming, which is manifested through the intention of green farming and finally transformed into the influence mechanism of behavioral intention acting on behaviors. Therefore, the interaction mechanism of the influencing factors on the green production behaviors of pig farmers is as follows: level of education → external pressure, farming conditions, operating characteristics → cognition of return → green production behavior of pig farmers, as shown in Figure 7.

## 5. Conclusions, Discussion and Recommendations

### 5.1. Conclusions

The Rational Peasant Theory and Prospect Theory are used in this paper to analyze pig farmers’ motivations for engaging in green production behaviors. Based on 747 questionnaires, this paper uses the Logit model and the ISM analysis method to explore the influencing factors (and their hierarchical structure) of the green production behavior of small and medium-sized pig farmers, and analyzes the mechanism of the influencing factors. The results show:

i. The green production behaviors of small-and medium-sized pig farmers are driven by the internal expected income, affected by the monitoring pressure from external stakeholders and constrained by their own resource capacity. In particular, the cognitive factors such as economic benefit, social benefit, growth benefit, social norm and breeding ability have significant effects on green production behavior.
ii. The influencing factors of different green production behaviors are different, and the influencing factors of scientific disease control, standardized management and waste recycling are more than that of rational feeding. Among them, cognition of economic benefit, social benefit, growth benefit, social norm, resource endowment, farming ability, farming profit, specialty and education level have a significant positive influence on the rationalization of feeding, and the breeding stock has a significant negative effect. Cognition of return, government regulation, organizational involvement, social norm, farming ability, education level, breeding stock and profit have a significant positive effect on the scientific epidemic control. Cognition of return, government regulation, organizational involvement, social norm, farming ability and education level have a significant positive effect on standardized management. Cognition of return, government regulation, social norm, farming ability, education level and profit have significant positive effects on manure recycling.
iii. The mechanism of different influencing factors on green production behavior is hierarchical. The cognition of return status of green production behavior is the direct factor; the external pressure, farming conditions and operating characteristics are the indirect factors; and the education level of decision makers is the underlying factor. Various factors are independent, interrelated and directly or indirectly act on the green production behavior of pig farmers.

### 5.2. Discussion

From the above results, the main hypotheses proposed in this paper are verified, but some variables fail to pass the test in different green production behaviors.

First of all, the cognition of expected benefit has a significant positive effect on the green production behaviors of small-and medium-sized pig farmers. In previous studies [10,15,31,32], expected benefits such as economic benefits, social benefits and environmental benefits have positive effects on green production behaviors, which is the same in this paper. In particular, growth income also has a significant impact on green production behaviors. However, the expected environmental benefits fail to pass the significance test, which shows that the small-and medium-sized farmers do not realize that reasonable feeding is an important measure to protect the ecological environment.

Secondly, the external pressure factors have a positive significant impact on the green production behaviors of small and medium-sized pig farmers. Previous study [7] has examined the role of environmental regulation on green production behavior, and this paper verifies this too. It is also found that community norms can promote green production behaviors. However, the influence factors of different green production behaviors are different, government regulation and consumption demand have no significant influence on input rationalization behavior, and consumption demand has no significant influence on waste recycling behavior.

Thirdly, the factor of resource endowment has a significant positive effect on the green production behaviors of small-and medium-sized pig farmers. Previous studies [40,41] have verified the role of resource endowment in the transformation from will to behavior; this paper also verifies this conclusion, especially the role of green production technology and equipment. However, this paper finds that land resources have little relevant on behaviors of scientific epidemic control, standardized management and manure resource.

Finally, regarding the control factors, contrary to what some previous studies [42,44] concluded, there is no significant relationship between age, sex or working hours and green production practices. Education level has a significant positive impact on green production behavior, which is consistent with the conclusion of the existing research [43].

Compared with the existing studies, this paper shows innovation in the following three aspects: First, it holds that the cognition of expected income serves as the primary motivator for the green production behaviors of small-and medium-sized pig farmers; in particular, growth benefits is tested, and it expands the results on the effect of expected return on green production behaviors. Secondly, the factors that contribute to green production behaviors are analyzed from material flows of pig farming, and the differences of the factors considered in different green production behaviors of the farmers are verified. The third step is investigating the mechanisms underlying the various factors that influence green production practices and confirming the hierarchy of these factors. This research deepens the existing studies by examining the mechanisms through which various factors interact.

### 5.3. Policy Suggestions

The following recommendations are made in light of the study’s findings in an effort to encourage pig farmers to adopt sustainable production practices:

First, strengthen policy support to maintain farmers’ expectations for returns on green production. Farmers’ expectations of return is the primary driver of green production practices; hence, all efforts should be made to raise these expectations. Since the 18th National Congress of the Communist Party of China (CPC), the environmental protection policies of pig farming have gradually tightened, and the health demand of residents has gradually increased. Farmers have begun to actively implement green production behaviors, and they have seen the financial gains, social effects and environmental benefits that come with green production—demonstrating the effectiveness of the prior policy support. So, it is necessary to: (i). keep strengthening government subsidies, maintaining long-term support policies such as subsidies for machinery purchases and land use approval, and raising farmers’ expectations for economic, social and environmental effects; (ii). continue to provide policy support for the construction of demonstration zones and farms, financial assistance for the construction of large-scale farms, and incentives for individual and small farmers to transition to standardized farms; and (iii). construct more farming infrastructure so that pig farms are well equipped with all necessary facilities, such as those for waste treatment, quality testing and the harmless treatment of sick and dead pigs, so as to lower the cost of green production.

Second, enhance oversight and public awareness to put pressure on pig farmers to carry out green production. External regulation is the intermediate factor for promoting green production behaviors; government regulations and social norms can be used to create a good atmosphere. It is suggested to (i). detail the supervision mechanism for green production. Local animal husbandry management departments shall detail the implementation standards and norms for disease control, environmental protection and other aspects to help farmers standardize their management. They shall intensify efforts to prevent and control epidemics and increase the frequency of inspections and penalties for pollutant discharge in order to urge green production practices. (ii). Give play to the supervision role of social groups and the general public to put pressure on farmers to adopt green production methods. iii. Strengthen guidance on consumer demand regarding the consumption of green pork and organic pork, and encourage farmers to conduct green food certification.

Third, strengthen the development of resource capacity to provide technical equipment support for farmers to embrace green farming practices. To do this, it is required to (i). innovate financial instruments, such as inclusive finance and digital finance, lowering the threshold of mortgage loans for financial institutions, and providing lending support for the transformation of green production methods for farmers; (ii). promote the combination of planting and farming, convert food crops to feed crops, promote the technical reform of feed engineering, and promote feeding rationalization; and (iii). urge financially strong farmers to carry out digital transformation, and guide small- and medium-sized farmers to implement green production practices by digital method.

Fourth, strengthen education and training, and firmly establish the green production awareness of pig farmers. To do it, it is suggested to (i). formulate preferential support policies and implement assistance programs to support the return of rural migrant workers and college students to develop green farming; (ii). strengthen the cultivation of farmers’ sense of social responsibility by vigorously promoting typical demonstration farms and strengthening technology and model exchanges; and (iii). cultivate a group of professional pig farming experts in order to make the pig farming and management process more standardized.

## Figures and Tables

**Table 1:** Green Production Behaviors of Pig Farmers and Specific Performances.

Green Production Behaviors	Specific Performances
Rationalization of feeding	Use biological fermented feed; add drugs or additives allowed for use in the feed; adopt improved pig breeds.
Scientific epidemic control	Traditional Chinese Medicine or biological veterinary medicine is used for prevention or treatment; regulations on the drug withdrawal period are observed; sick and dead pigs are treated innocuously; pig houses and delivery vehicles are disinfected regularly.
Standardized management	The farms are standard or upgraded; farming files such as medication records are consistently kept; farms are frequently cleaned and sterilized.
Manure recycling	There are special manure storage facilities, biogas digesters, or manure treatment equipment; biological fermentation treatment technology is used; the manure is returned to the field.

**Table 2:** Assignment and Description of Variables.

Variables	Name	Definition	Assignment	Mean	Standard Deviation	Expected Impact
Explained variables						
Green production behaviors	Rationalization of feeding	See Table 1 for details	1 = at least one answer is yes; 0 = all answers are no.	0.70	0.46	
	Scientific epidemic control			0.70	0.46	
	Standardized management			0.76	0.43	
	Manure recycling			0.77	0.42	
Explanatory variables						
Cognition of return	Economic benefits	Green production behaviors can increase economic income; Green production behaviors can save farming costs.	Completely disagree = 1; Comparatively disagree = 2; Generally = 3; Comparatively agree = 4; Completely agree = 5	3.69	1.23	+
	Social benefits	Green production behaviors can improve product quality; Green production behaviors can improve product quality.		3.67	1.20	+
	Environmental benefits	Green production behaviors can improve the quality of ecological environment; Green production behaviors can reduce pollution emissions.		3.64	1.32	+
	Growth benefits	Green production behaviors can promote employee growth; Green production behaviors can regulate internal management		3.66	1.28	+
External pressure	Government regulation	The government has strict requirements for green farming		3.62	1.14	+
	Consumer demand	Consumers have the higher requirements for quality		3.66	1.13	+
	Social norms	Social norms have strict supervision over green farming		3.63	1.20	+
Resource capacity	Resource endowment	Whether there is arable or forest land	1 = Yes; 0 = No	0.59	0.49	+
	Farming capacity	Automation technology applied (including feeding, drinking, cleaning manure, etc.)	Not applied = 1; One applied = 2; Two applied = 3; Three applied = 4; More than four applied = 5	3.59	1.28	+
Individual characteristics	Gender	Sex of farm owner	Male = 1; Female = 0	0.91	0.28	+
	Age	Age of farm owner	Actual age (years)	49.34	8.92	−
	Level of education	Level of education of farm owners	Primary school and below = 1; junior high school = 2; senior high school = 3; college and undergraduate = 4; graduate student and above = 5	1.97	1.06	+
	Specialty	Owner has studied relevant specialties	1 = Yes; 0 = No	0.43	0.50	+
Operating characteristics	Farming time	Farming years of the farm	Actual farming period (years)	12.9	6.56	+
	Breeding stock	Number of columns in the previous year	Year-end farm stock (PCS)	102	137.9	+
	Farming profit	Breeding stock of the previous year	Actual profit of the farm at the end of the year (ten thousand yuan)	115	253.1	+

**Table 3:** Description of Sample Characteristics.

Characteristics	Classification	Overall		Individual Pig Farmers		Small-Sized		Medium-Sized	
		Occurrence Number	Frequency%	Occurrence Number	frequency%	Occurrence Number	Frequency%	Occurrence Number	Frequency%
Gender	Male	683	91.4	149	88.2	377	92.5	157	92.3
	Female	64	8.6	20	11.8	31	7.5	13	7.7
Age	≤30 years old	9	1.2	1	0.6	5	1.2	3	1.8
	30–39 years old	87	11.6	19	11.2	49	12	19	11.2
	40–49 years old	247	33.1	60	35.5	130	31.8	57	33.5
	50–59 years old	315	42.2	67	39.6	176	43.1	72	42.4
	≥60 years old	89	12	22	13	48	11.8	19	11.2
Level of education	Primary school and below	88	11.6	16	9.4	58	14.2	14	8.2
	Junior high	361	48.3	72	42.6	202	49.5	87	51.2
	High school (technical secondary school)	231	30.1	65	38.4	117	28.7	49	21.8
	College and undergraduate	67	9	16	9.4	31	7.6	20	11.8
	Master degree and above	0	0	0	0	0	0	0	0
Concurrent business degree	Full-time farming	457	61.2	96	56.8	254	62.3	107	62.9
	Semi-farming	253	33.9	60	35.5	135	33.1	58	34.1
	Part-time farming	37	4.9	13	7.7	19	4.7	5	2.9

**Table 4:** Regression Results of Influencing Factors of Different Green Production Behaviors of Pig Farmers.

		Rationalization of Feeding		Scientific Epidemic Control		Standardized Management		Manure Recycling	
Variable Classification	Name	Model 1	Model 2	Model 1	Model 2	Model 1	Model 2	Model 1	Model 2
Cognition of return	Cognition of economic benefits	0.814 *** (0.000)	0.865 *** (0.000)	0.986 *** (0.001)	0.865 *** (0.002)	0.470 ** (0.011)	0.447 *** (0.008)	0.546 *** (0.003)	0.584 *** (0.001)
	Cognition of social benefits	1.418 ** (0.000)	1.207 *** (0.000)	0.937 *** (0.008)	0.895 *** (0.000)	0.580 *** (0.004)	0.503 *** (0.008)	1.676 *** (0.000)	1.609 *** (0.000)
	Cognition of environmental benefits	0.245 (0.245)	——	0.519 * (0.086)	0.482 * (0.089)	0.447 ** (0.013)	0.494 *** (0.003)	0.769 *** (0.000)	0.742 *** (0.000)
	Cognition of growth benefits	0.851 *** (0.000)	0.880 *** (0.000)	1.039 *** (0.001)	0.890 *** (0.001)	0.535 *** (0.004)	0.449 *** (0.008)	0.496 *** (0.007)	0.476 ** (0.013)
External pressure	Government regulation	0.083 (0.741)	——	1.706 *** (0.000)	1.661 *** (0.000)	0.503 ** (0.028)	0.511 ** (0.012)	0.470 * (0.070)	0.455 * (0.050)
	Consumer demand	0.323 (0.181)	——	2.161 *** (0.000)	2.145 *** (0.000)	0.488 * (0.091)	0.613 ** (0.018)	0.022 (0.943)	——
	Social norms	1.205 *** (0.000)	1.414 *** (0.000)	0.831 ** (0.019)	0.723 ** (0.034)	0.985 *** (0.000)	0.897 *** (0.000)	0.878 *** (0.002)	0.859 *** (0.002
Resource capacity	Resource endowment	1.026 *** (0.001)	1.041 *** (0.001)	0.031 (0.994)	——	0.057 (0.869)	——	0.301 (0.404)	——
	Farming capacity	0.351 ** (0.039)	0.390 ** (0.024)	0.659 * (0.056)	0.630 * (0.050)	0.555 *** (0.002)	0.631 *** (0.000)	0.313 * (0.082)	0.239 * (0.089)
Individual characteristics	Sex of owner	0.110 (0.787)	——	0.527 (0.664)	——	−0.312 (0.525)	——	−0.164 (0.773)	——
	Age of the owner	0.024 (0.222)	——	0.039 (0.333)	——	−0.005 (0.840)	——	0.019 (0.421)	——
	Level of education	0.811 *** (0.000)	0.866 *** (0.000)	2.359 *** (0.000)	1.875 *** (0.001)	0.860 ** (0.044)	1.292 *** (0.000)	0.711 ** (0.016)	0.448 ** (0.049)
	Expertise	1.437 *** (0.000)	1.390 *** (0.000)	1.205 * (0.051)	——	0.149 (0.756)	——	0.644 (0.167)	——
Operating characteristics	Working time	−0.023 (0.293)	——	0.045 (0.324)	——	0.044 (0.110)	——	0.047 (0.118)	——
	Breeding stock	−0.004 *** (0.001)	−0.004 *** (0.002)	0.054 *** (0.000)	0.056 *** (0.000)	0.022 (0.122)	——	0.001 (0.777)	——
	Farming profit	0.048 * (0.058)	0.049 * (0.056)	0.128 *** (0.000)	0.128 *** (0.000)	0.035 (0.802)	——	0.015 * (0.061)	0.019 *** (0.001)
	Constant term	−7.235 *** (0.000)	−5.773 *** (0.000)	−24.84 *** (0.000)	−21.01 *** (0.000)	−9.409 *** (0.000)	−9.507 *** (0.000)	−11.22 *** (0.000)	−9.491 *** (0.000)
	Adjusted R^2^	0.589	0.581	0.863	0.859	0.692	0.664	0.695	0.683
	Significance *p*-value	0.000	0.000	0.000	0.000	0.000	0.000	0.000	0.000

Note: the values in brackets are *p* values, *** means *p* < 0.01, ** means *p* < 0.05, and * means *p* < 0.1.

## Data Availability

Not applicable.

## References

[1] Gan L., Hu X.S. (2016). The pollutants from livestock and poultry farming in China—Geographic distribution and drivers. Environ. Sci. Pollut. Res..

[2] Nguyen V.H., Dang X.S., Pham D.P., Roesel K., Huong N.M., Luu-Quoc T., Van Hung P., Thi Duong Nga N., Lapar L., Unger F. (2019). Rapid integrated assessment of food safety and nutrition related to pork consumption of regular consumers and mothers with young children in Vietnam. Glob. Food Secur..

[3] Zhang D., Wang X., Zhou Z. (2017). Impacts of small-scale industrialized swine farming on local soil, water and crop qualities in a hilly red soil region of subtropical China. Int. J. Environ. Res. Public Health.

[4] Wilk J., Andersson L., Warburton M. (2013). Adaptation to climate change and other stressors among commercial and small-scale South African farmers. Reg. Environ. Change.

[5] Huang Z.H., Zhong Y.Q., Wang X.L. (2016). Effects of different policies on farmers’ pesticide application behavior. China Popul. Resour. Environ..

[6] Wang J., Chen K., Wu L., Zhao P., Li M. (2020). Factors affecting the willingness of agricultural green production from the perspective of farmers’ perceptions. Sci. Total Environ..

[7] Li M., Liu Y., Huang Y., Wu L., Chen K. (2022). Impacts of Risk Perception and Environmental Regulation on Farmers’ Sustainable Behaviors of Agricultural Green Production in China. Agriculture.

[8] Qiao D., Xu S., Xu T., Hao Q., Zhong Z. (2022). Gap between Willingness and Behaviors: Understanding the Consistency of Farmers’ Green Production in Hainan, China. Int. J. Environ. Res. Public Health.

[9] Du S., Liu J., Fu Z. (2021). The impact of village rules and formal environmental regulations on farmers’ cleaner production behavior: New evidence from China. Int. J. Environ. Res. Public Health.

[10] Gholamrezai S., Aliabadi V., Ataei P. (2021). Understanding the pro-environmental behavior among green poultry farmers: Application of behavioral theories. Environ. Dev. Sustain..

[11] Teng Y., Pang B., Zhang M., Guo X. (2022). Driving mechanism of farmers’ green production behavior under normalization of COVID-19 prevention and control: A case study in China. Front. Public Health.

[12] Zhong Y., Tang L., Li Y. (2022). Role of Digital Empowerment in Developing Farmers’ Green Production by Agro-Tourism Integration in Xichong, Sichuan. Agriculture.

[13] Li C., Shi Y., Khan S.U., Zhao M. (2021). Research on the impact of agricultural green production on farmers’ technical efficiency: Evidence from China. Environ. Sci. Pollut. Res..

[14] Mao H., Chai Y., Chen S. (2021). Land Tenure and Green Production Behavior: Empirical Analysis Based on Fertilizer Use by Cotton Farmers in China. Int. J. Environ. Res. Public Health.

[15] Adnan N., Nordin S.M., Bahruddin M.A., Tareq A. (2019). A state-of-the-art review on facilitating sustainable agriculture through green fertilizer technology adoption: Assessing farmers behavior. Trends Food Sci. Technol..

[16] Li Y., Fan Z., Jiang G., Quan Z. (2021). Addressing the Differences in Farmers’ Willingness and Behavior Regarding Developing Green Agriculture—A Case Study in Xichuan County, China. Land.

[17] Ding J., Li B., Zhang B., Wang J., Zhang L. (2021). Key factors affecting the adoption willingness, behavior, and willingness-behavior consistency of farmers regarding photovoltaic agriculture in China. Energy Policy.

[18] United Nations Environment Programme (2011). Towards a Green Economy: Pathways to Sustainable Development and Poverty Eradication.

[19] Paul I.D., Bhole G.P., Chaudhari J.R. (2014). A review on green manufacturing: It’s important, methodology and its application. Procedia Mater. Sci..

[20] He Z.J., Wang J.M., Ma H.Y., Zhang Z.H., Zhao M.Z. (2020). Why the gap between the production costs of agricultural products in China and the United States is growing: The case of hogs. Issues Agric. Econ..

[21] Hu Y., Yu Y. (2022). Scale Difference from the Impact of Disease Control on Pig Production Efficiency. Animals.

[22] Li X., Wang M., Xue Y., Duan D., Li C., Ye J., Han X., Qiao R., Wang K., Li X. (2021). Characterization and comparison of the bacterial community between complete intensive and extensive feeding patterns in pigs. AMB Express.

[23] Wang B., Dong F., Chen M., Zhu J., Tan J., Fu X., Wang Y., Chen S. (2016). Advances in recycling and utilization of agricultural wastes in China: Based on environmental risk, crucial pathways, influencing factors, policy mechanism. Procedia Environ. Sci..

[24] Atanu S., Love H.A., Schwart R.B. (1994). Adoption of emerging technologies under output uncertainty. Am. J. Agric. Econ..

[25] Kong X.Z., Fang S.H., Pang X.P. (2004). Analysis of the impact of farm household endowments on agricultural technology adoption in western China. Econ. Res. J..

[26] Camerer C.F. (2004). Prospect theory in the wild: Evidence from the field. Advances in Behavioral Economics.

[27] Kahneman D., Tversky A. (2013). Prospect theory: An analysis of decision under risk. Handbook of the Fundamentals of Financial Decision Making: Part I.

[28] Foster A.D., Rosenzweig M.R. (2010). Microeconomics of technology adoption. Annu. Rev. Econ..

[29] McGlone J.J. (2013). The future of pork production in the world: Towards sustainable, welfare-positive systems. Animals.

[30] Teng Y., Chen X., Yu Z., Wei J. (2021). Research on the Evolutionary Decision-Making Behavior Among the Government, Farmers, and Consumers: Based on the Quality and Safety of Agricultural Products. IEEE Access.

[31] Ataei P., Gholamrezai S., Movahedi R., Aliabadi V. (2021). An analysis of farmers’ intention to use green pesticides: The application of the extended theory of planned behavior and health belief model. J. Rural Stud..

[32] Savari M., Zhoolideh M., Khosravipour B. (2021). Explaining pro-environmental behavior of farmers: A case of rural Iran. Curr. Psychol..

[33] Wang J., Shen M., Chu M. (2021). Why is green consumption easier said than done? Exploring the green consumption attitude-intention gap in China with behavioral reasoning theory. Clean. Responsible Consum..

[34] Qiao D., Luo L., Zheng X., Fu X. (2022). External Supervision, Face Consciousness, and Pesticide Safety Use: Evidence from Sichuan Province, China. Int. J. Environ. Res. Public Health.

[35] Li G., Wang X., Wu J. (2019). How scientific researchers form green innovation behavior: An empirical analysis of China’s enterprises. Technol. Soc..

[36] Aceleanu M.I. (2016). Sustainability and competitiveness of Romanian farms through organic agriculture. Sustainability.

[37] Wang J., Deng Y., Diao H. (2018). Market returns, external pressure, and safe pesticide practice—Moderation role of information acquisition. Int. J. Environ. Res. Public Health.

[38] Bolnick D.I., Svanbäck R., Fordyce J.A., Yang L.H., Davis J.M., Hulsey C.D., Forister M.L. (2003). The ecology of individuals: Incidence and implications of individual specialization. Am. Nat..

[39] Luo L., Qiao D., Tang J., Wan A., Qiu L., Liu X., Liu Y., Fu X. (2022). Training of Farmers’ Cooperatives, Value Perception and Members’ Willingness of Green Production. Agriculture.

[40] Lu H., Xie H. (2018). Impact of changes in labor resources and transfers of land use rights on agricultural non-point source pollution in Jiangsu Province, China. J. Environ. Manag..

[41] Ren J., Li F., Yin C., Zhang J. (2022). Uncovering the Deviation of Farmers’ Green Manure Planting Willingness and Behavior. Sustainability.

[42] Elahi E., Khalid Z., Zhang Z. (2022). Understanding farmers’ intention and willingness to install renewable energy technology: A solution to reduce the environmental emissions of agriculture. Appl. Energy.

[43] Guo H., Sun F., Pan C., Yang B., Li Y. (2021). The deviation of the behaviors of rice farmers from their stated willingness to apply biopesticides—A study carried out in Jilin Province of China. Int. J. Environ. Res. Public Health.

[44] Luo L., Qiao D., Tang J., Wang L., Liu Y., Fu X. (2022). Research on the influence of education and training of farmers’ professional cooperatives on the willingness of members to green production—Perspectives based on time, method and content elements. Environ. Dev. Sustain..

[45] Gao Y., Zhao D., Yu L., Yang H. (2019). Duration analysis on the adoption behavior of green control techniques. Environ. Sci. Pollut. Res..

[46] Wang H., Zhong S., Guo J., Fu Y. (2021). Factors affecting green agricultural production financing behavior in Heilongjiang family farms: A structural equation modeling approach. Front. Psychol..

[47] Sun S.M., Zhang Y.Y., Zhang J.R. (2012). An empirical analysis of factors influencing willingness to implement good quality and safety practices in pig farms (households) based on Logit-ISM model. China Rural Econ..

[48] Warfield J.N. (1978). Societal systems planning, policy and complexity. Cybern. Syst..

